# Thermal Response of* In Vivo* Human Skin to Fractional Radiofrequency Microneedle Device

**DOI:** 10.1155/2016/6939018

**Published:** 2016-05-09

**Authors:** Woraphong Manuskiatti, Penvadee Pattanaprichakul, Siriluk Inthasotti, Panitta Sitthinamsuwan, Suchanan Hanamornroongruang, Rungsima Wanitphakdeedecha, Sorawuth Chu-ongsakol

**Affiliations:** ^1^Department of Dermatology, Faculty of Medicine Siriraj Hospital, Mahidol University, Bangkok 10700, Thailand; ^2^Department of Pathology, Faculty of Medicine Siriraj Hospital, Mahidol University, Bangkok 10700, Thailand; ^3^Division of Plastic Surgery, Department of Surgery, Faculty of Medicine Siriraj Hospital, Mahidol University, Bangkok 10700, Thailand

## Abstract

*Background*. Fractional radiofrequency microneedle system (FRMS) is a novel fractional skin resurfacing system. Data on thermal response to this fractional resurfacing technique is limited.* Objectives*. To investigate histologic response of* in vivo* human skin to varying energy settings and pulse stacking of a FRMS in dark-skinned subjects.* Methods*. Two female volunteers who were scheduled for abdominoplasty received treatment with a FRMS with varying energy settings at 6 time periods including 3 months, 1 month, 1 week, 3 days, 1 day, and the time immediately before abdominoplasty. Biopsy specimens were analyzed using hematoxylin and eosin (H&E), Verhoeff-Van Gieson (VVG), colloidal iron, and Fontana-Masson stain. Immunohistochemical study was performed by using Heat Shock Protein 70 (HSP70) antibody and collagen III monoclonal antibody.* Results*. The average depth of radiofrequency thermal zone (RFTZ) ranged from 100 to 300 *μ*m, correlating with energy levels. Columns of cell necrosis and collagen denaturation followed by inflammatory response were initially demonstrated, with subsequent increasing of mucin at 1 and 3 months after treatment. Immunohistochemical study showed positive stain with HSP70.* Conclusion*. A single treatment with a FRMS using appropriate energy setting induces neocollagenesis. This wound healing response may serve as a mean to improve the appearance of photodamaged skin and atrophic scars.

## 1. Introduction

The concept of fractional photothermolysis (FP) has been developed to overcome the limitations of ablative and nonablative resurfacing systems [1]. Subsequently, FP has been considered as a new technique for facial rejuvenation by inducing homogeneous, fractional thermal damage at a particular depth of the skin, followed by collagen remodeling process. This technique creates microscopic noncontiguous columns of thermal injury in the dermis (referred to as microscopic thermal zones or MTZ), surrounded by zones of viable tissue. Therefore, the reepithelization process can occur faster with fewer side effects, when comparing to those of ablative skin resurfacing system [2–4].

Application of fractional technology to nonablative skin rejuvenation techniques has potential to provide efficacy while maintaining advantages of minimal to no downtime. However, despite these safety advantages the clinical efficacy profile does not match that of the full ablative process, especially with respect to moderate-to-severe rhytides and atrophic scars. Subsequently, fractional delivery systems for ablative lasers, including CO_2_ and Er:YAG, have also been developed in an attempt to match the clinical results achieved with traditional ablative lasers. Ablative fractional lasers (AFLs) appear to lead to a more robust wound healing response and accompanying dermal fibrosis, which may explain the rapid and significant clinical effects that can be achieved with ablative versus nonablative devices. However, AFLs are associated with a higher risk of postinflammatory hyperpigmentation (PIH), compared with that of nonablative fractional lasers (NAFLs) [4, 5].

Fractional radiofrequency (RF) system is one of novel fractional resurfacing techniques. It creates controlled thermal damage in the dermis and stimulates wound healing response, initiating collagen remodeling process by using thermal production from tissue impedance and subsequent heat diffusion to deeper tissue. An advantage of fractional radiofrequency is that it causes less epidermal disruption by only 5%, compared with 10–70% of that of fractional ablative laser systems [6]. The healing process is faster with minimal downtime. Therefore, this technique may be an alternative choice of facial rejuvenation and atrophic scars, especially in patients with darker skin complexion.

Recently, fractional radiofrequency microneedle system (FRMS) was introduced [7, 8]. By using insulated microneedle electrodes penetrating through the epidermis to directly deliver radiofrequency pulse at the upper dermal level while sparing the epidermis, this technique has shown to provide equivalent efficacy with less side effects, comparing with traditional ablative fractional resurfacing procedures [9].

The current available information on the wounding response of human skin to FRMS treatment is limited. Several of the previous studies on wound healing responses to FRMS only investigated in the Western subjects [3, 10]. In fact, racial differences in skin pathophysiology have been well documented [11]. The high risk of pigmentary alterations and scarring following any procedure that produces inflammation of the skin continues to influence physicians to exercise caution with this group of patients.

The purpose of this study was to investigate the histological response after FRMS treatment with varying energy settings and pulse stacking techniques in dark-skinned individuals by using histopathology and immunohistochemistry to identify zones of denatures collagen, inflammatory infiltration, neoelastogenesis, and neocollagenesis, as well as any side effects to pigmentation and textural alteration, throughout the 3-month study.

## 2. Materials and Methods

### 2.1. Study Design

Two female participants (skin phototype IV), aged 43 and 45 years, prescheduled for abdominoplasty by a plastic surgeon, were enrolled in the study. Both patients had no underlying skin disease, no history of topical medication use within 1 month, and no history of laser treatment within 6 months on their abdomen skin prior to the study. This study was approved by the Siriraj Institutional Review Board (SIRB) and written informed consent was obtained from all study participants.

### 2.2. Fractional Radiofrequency Device

Study subjects were treated with a FRMS (INTRAcel system, Jeisys Inc., Korea). The handpiece of this system consisted of 49-gauge needle electrodes in a 1 × 1 cm^2^ area. Each electrode was 1.5–2.0 mm in length. While the proximal 0.5 mm of each electrode was insulated with a biocompatible material, the uncovered distal part emitted RF pulse to the treated area. Treatment levels can be adjusted ranging from the test level to level 5 (Table 1). The study subjects were treated with different energy levels at 6 different time periods, including immediately, 1 day, 3 days, 1 week, 1 month, and 3 months prior to their prescheduled abdominoplasty to follow up on the chronological evolution of the* in vivo* wounding responses (Table 2).

### 2.3. Treatment Regimens

**Figure 1:**
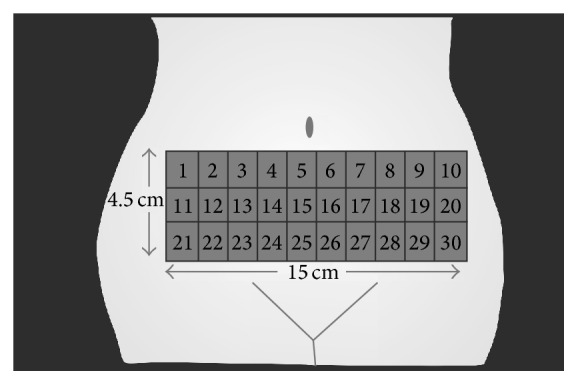
Illustration showing 30 of 1.5 × 1.5 cm^2^ squares, tattooed on each patient's abdominal wall.

Prior to surgery, 30 squares, size 1.5 × 1.5 cm^2^, were tattooed on each patient's abdominal wall as shown in Figure 1. The study participants were scheduled to receive 6 treatment sessions, immediately, 1 day, 3 days, 1 week, 1 month, and 3 months before their prescheduled abdominoplasty. At each visit, each participant received FRMS treatment using 5 different energy levels on 5 test areas (Table 2). Preoperatively, the treated area was cleaned with 2% chlorhexidine solution. Lidocaine 2.5% and prilocaine 2.5% cream (a eutectic mixture of local anesthetic, AstraZeneca LP, Wilmington, DE) was applied under occlusion for an hour before treatment.

Immediately before abdominoplasty, 30 biopsy specimens from the marked test sites and one from a controlled untreated site were collected by using a 4 mm punch biopsy (Stiefel Laboratories, NC, USA). Each of the specimens was placed into 10% (v/v) neutral buffered formalin overnight before paraffin embedding. The specimens were sectioned vertically into 5 *μ*m thick slices and stained with hematoxylin and eosin (H&E), Verhoeff-Van Gieson (VVG) elastic, colloidal iron, and Masson-Fontana stain for histopathologic analysis.

Immunohistochemical study was performed by using Heat Shock Protein 70 (HSP70) antibody (Thermo Scientific, MA, USA) for evaluation of inflammatory response and neocollagenesis. The staining method for HSP70 was done in the following steps. Antigen retrieval was performed by incubation in citrate buffer (pH 6.0) at 95°C for 40 minutes in water bath and neutralized endogenous peroxidase activity with 3% hydrogen peroxidase followed by block nonspecific binding with 2% bovine serum albumin for 20 minutes [12]. The histological examination of each specimen was evaluated by using light microscope and was further recorded by using a DP 72 microscope digital camera (Olympus Corporation, Tokyo, Japan).

### 2.4. Outcome Measures

The zones of denatured collagen or radiofrequency thermal zone (RFTZ) and neocollagenesis of all specimens were evaluated by 2 experienced dermatopathologists (Penvadee Pattanaprichakul and Suchanan Hanamornroongruang) who were blinded to the corresponding treatment protocol and tissue labeling. The effects of varying energy settings and pulse stacking on the depth of RFTZ and neocollagenesis were described.

Side effects associated with each treatment such as hypo- and hyperpigmentation, pinpoint bleeding, purpura, and textural change were recorded.

## 3. Results

### 3.1. Depth of RFTZ

The average depth of RFTZ ranged from 100 to 300 *μ*m, correlating with energy levels. The maximum depth of RFTZ was 300 *μ*m at a site treated with energy level 5 (80 watts, wave duration of 50 milliseconds (ms), and electrode insertion duration of 300 ms), whereas the minimum depth of RFTH was 100 *μ*m at a site treated with energy level 2 (50 watts, wave duration of 50 milliseconds (ms), and electrode insertion duration of 300 ms). RFTZ was not detectable on a site treated with test level (electrode insertion only).

**Figure 2:**
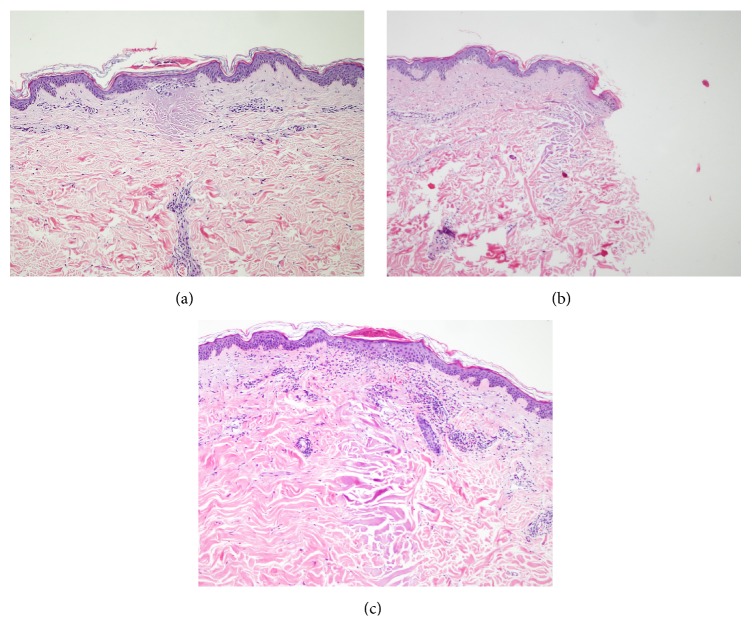
Demonstration of confined zone of denatured collagen or radiofrequency thermal zone (RFTZ), immediately after treatment. (a) RFTZ is initially found at treatment level 2 (50 watts, wave duration of 50 milliseconds (ms), and electrode insertion duration of 300 ms). (b) RFTZ at treatment level 4 (50 watts, wave duration of 100 milliseconds (ms), and electrode insertion duration of 350 ms). (c) RFTZ at treatment level 5 (80 watts, wave duration of 50 milliseconds (ms), and electrode insertion duration of 300 ms), H&E stain; original magnifications: 10x.

**Figure 3:**
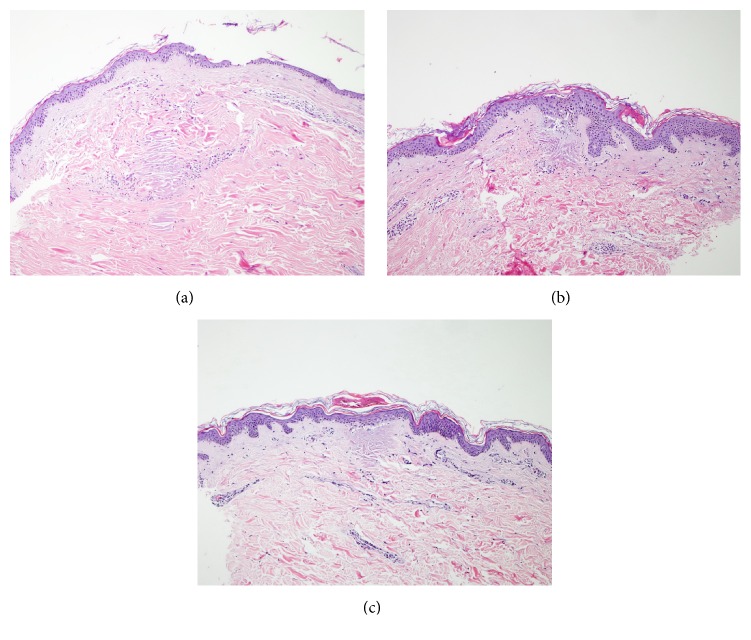
Demonstration of tissue response following treatment. (a) The inflammatory cells are found within 24 hours around RFTZs at 1 day after treatment. (b) Three days. (c) One week. (H&E stain; original magnifications: 10x.)

Figure 2 demonstrates confined zone of denatured collagen or radiofrequency thermal zone (RFTZ). The confined zone of denatured collagen from the specimens was demonstrated in the specimens treated with energy starting from level 2 (50 watts, wave duration of 50 milliseconds (ms), and electrode insertion duration of 300 ms). Inflammatory cell infiltrations were also noted. However, this inflammatory response was disappeared by 3 months following treatment in both patients (Figure 3). There was no evidence of collagen denaturation observed at the site treated with electrode insertion alone and the site treated with energy level 1 (50 watts, wave duration of 30 milliseconds (ms), and electrode insertion duration of 280 ms).

### 3.2. Neocollagenesis

In order to identify the evidence of neocollagenesis, HSP70 immunohistochemical study was used to investigate wound healing cascade. HSP70 staining started showing positive staining at superficial perivascular inflammatory cells at energy level 2 (50 watts, wave duration of 50 milliseconds (ms), and electrode insertion duration of 300 ms) by 1 day after treatment, peaked at 7 days, and was not evident at 1 month and beyond. A finding of dermal coagulation zone, the presence of inflammatory response (positive staining of HSP70), together with detection of dermal mucin production (positive colloidal iron stain), confirmed an evidence toward neocollagenesis.

### 3.3. Inflammatory Cells

Tissue response to FRMS through 6 time periods was demonstrated in Figure 3. Within the first 24 hours after treatment, predominant neutrophils infiltration around RFTZ was observed. Lymphocytic infiltration was found as early as 3 days after treatment and persisted up to 1 month.

### 3.4. Mucin Production

**Figure 4:**
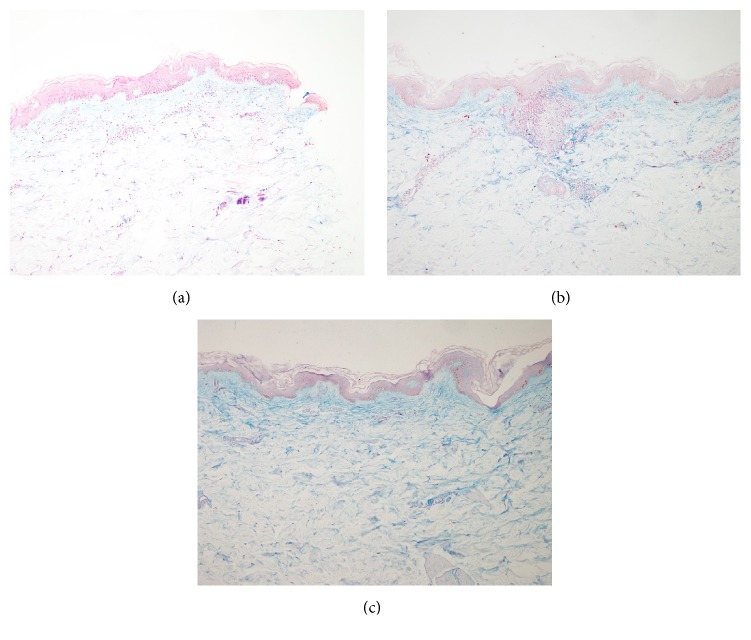
Demonstration of increasing amount of mucin around RFTZ starting at 1 month after treatment. (a) One week. (b) One month after treatment. (c) Three months. (Colloidal iron stain; original magnifications: 10x.)

The amounts of mucin at 1 month and 3 months were increased, compared with that of the untreated site, demonstrated by colloidal iron stain. The results are shown in Table 3 and Figure 4.

### 3.5. Neoelastogenesis

**Figure 5:**
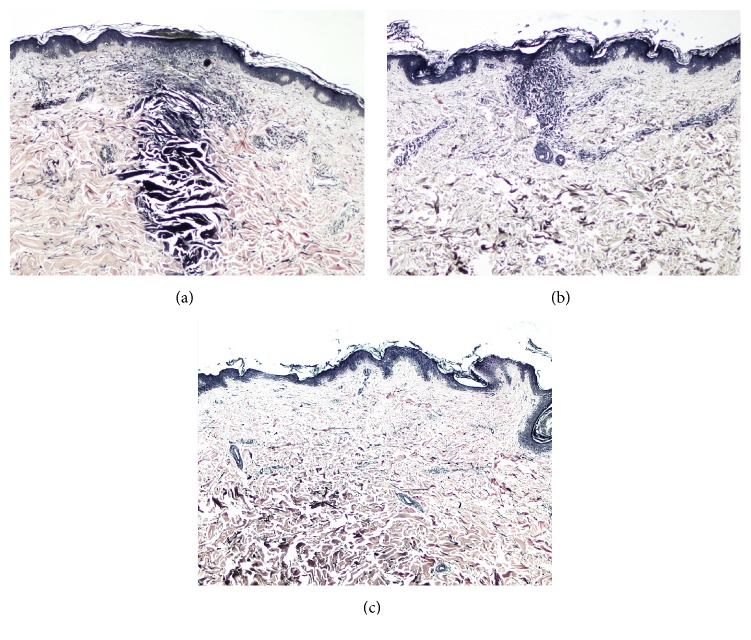
Neoelastogenesis. (a) Immediately after treatment, enlarged and clumped elastic fibers were found, representing denatured elastic fibers from heat. (b) There is increasing amount of smaller size elastic fibers around zones of denatured collagen compared to nontreated site at 1 month after treatment. (c) Three months after treatment. (Verhoeff-Van Gieson stain; original magnifications: 10x.)

The well-formed elastic fibers were also increased at 1 and 3 months after treatment demonstrated by positive stain of VVG (Figure 5).

### 3.6. Melanin Pigment

The density of melanin incontinence gradually decreased from the time immediately after treatment until it became completely disappeared at the 3-month follow-up visit.

### 3.7. Adverse Effect

Pinpoint bleeding was observed immediately after treatment and stopped after compression for 2-3 minutes. Mild hyperpigmentation at the test sites treated with energy level 5 in both subjects was the only adverse effect observed in this study. This pigmentary change was observed at 1 month after treatment and disappeared at the 3-month follow-up visit.

## 4. Discussion

The present study demonstrated the potential application of FRMS to induce neocollagenesis by creating multiple microscopic zones of denatured collagen or radiofrequency thermal zone (RFTZ) intervening with normal tissue in the dermis as the result of the fractional thermal response. In addition, the current study showed that the depth of RFTZ could be controlled by the radio wave duration and time of electrodes existing in the skin. The formation of denatured collagen zones was not detectable at the site treated with electrode insertion alone and the site treated with low energy level (energy level 1). However, zone of collagen denaturation was initially observed at energy level 2 (50 watts, wave duration of 50 milliseconds (ms), and electrode insertion duration of 300 ms). This observation implies that an induction of neocollagenesis requires an optimum level of dermal heating and physical insertion of the electrode only without a sufficient thermal influence may not be able to stimulate a process of new collagen formation.

Recently, numerous studies have shown the protective effects of HSPs on different organs subjected to different environmental stress including radiation, oxidative damage, heavy metals, and thermal stress. In skin, cells of epidermis and dermis express HSPs, which confer resistance to damage caused by stressors [13, 14]. The heat shock response of cells is thought to have a role in ameliorating cellular injury and preventing programmed cell death. The protective effects of HSPs are likely mediated by the ability of these proteins to function as molecular chaperones, preventing inappropriate protein aggregation and facilitating transport of immature proteins to target organelles for packing, degradation, or repair [15]. However, these proteins show different expression patterns depending on the types of stressor and cell. Earlier studies have shown that intense pulsed light (IPL) and laser treatments induce expression of several Heat Shock Proteins (HSPs) including HSP47, HSP70, and HSP72 by dermal dendritic cells [16, 17]. It is believed that activation of these cells may be the underlying mechanism of collagen deposition [3, 15]. Similar to a finding of the present study, an expression of HSP70 was detectable following Nd:YAG [18] and CO_2_ [19] laser irradiation. Additionally, a previous study revealed that the extent and duration of HSP70 induction were varied with laser pulse durations and radiant exposures [19].

Zones of denatured collagen had been replaced by newly formed collagen fiber by 3 months after a single FRMS treatment. This corresponds with the presence of an increasing amount of mucin deposition surrounding RFTZs from 1- to 3-month follow-up visits. In addition, an increased amount of well-formed elastic fiber, representing neoelastogenesis, was demonstrated by Verhoeff-Van Gieson stain at 1- and 3-month follow-up visit. Similar to the present study, earlier studies on FRMS treatment used a fractional radiofrequency device with a different parameter setting to create fractionated tissue response [8, 20]. Both neoelastogenesis and neocollagenesis have been demonstrated following FRMS treatment. However, most of the study subjects of the aforementioned studies had skin phototypes I-II and were followed up for a shorter period of time, compared to our study. Taken together, the evidences of the present study suggest that thermal response to FRMS treatment induces wound healing cascade leading to long-term dermal remodeling with increased collagen synthesis.

Hyperpigmentation was the only adverse effect observed in both patients (100%) on the area treated by using high energy level and resolved by 3 months after treatment. This finding correlated with the results from melanin staining, demonstrating moderate amounts of melanin pigments immediately after treatment that nearly disappeared by 3 months. A previous study using a similar bipolar microneedle radiofrequency device in patients with a majority of skin phototypes II and III reported no evidence of PIH in all 15 subjects treated. The physician should be aware that hyperpigmentation can develop as an adverse effect following a FRMS treatment, especially in dark-skinned patients. A limitation of the present study was the small number of subjects (*n* = 2). However, the histopathological and immunohistochemistry findings in both subjects were identical.

In summary, the present study demonstrated an evidence of neocollagenesis following microscopic thermal heating to the dermis by using FRMS at an optimum heating level. Further studies, enrolling a larger number of volunteers, are required to optimize parameter setting for maximizing clinical efficacy and minimizing adverse effects.

## Figures and Tables

**Table 1:** Treatment parameters.

Energy	Voltage (watt)	Radio wave (millisecond)	Time of electrodes existing in the skin (millisecond)
Test level (needle insertion only)	0	0	250
1	50	30	280
2	50	50	300
3	50	80	330
4	50	100	350
5	80	50	300

**Table 2:** Sequenced treatment plan of all test areas illustrated in Figure 1.

Visiting time (before surgery)/parameters	3 mo	1 mo	1 wk	3 d	1 d	Imm
Test level (needle insertion only) 1 pass	1	6	11	16	21	26
Level 2 1 pass	2	7	12	17	22	27
Level 2 2 passes	3	8	13	18	23	28
Level 4 1 pass	4	9	14	19	24	29
Level 5 1 pass	5	10	15	20	25	30

Imm = immediately before abdominoplasty, d = day, wk = week, and mo = month.

**Table 3:** Qualitative grading of mucin production after treatment.

Energy	Imm	1 d	3 d	1 wk	1 mo	3 mo
Test level	0	0	0	0	0	0
2	0	0	0	0	+	+
2/2 passes	0	0	0	0	++	++
4	0	0	0	0	++	++
5	0	0	0	0	++	++

Imm = immediately before abdominoplasty, d = day, wk = week, mo = month, 0 = no significant staining detected, + = minimally positive staining, and ++ = moderately positive, compared to nontreated site.
